# *INHBA* is Enriched in HPV-negative Oropharyngeal Squamous Cell Carcinoma and Promotes Cancer Progression

**DOI:** 10.1158/2767-9764.CRC-23-0258

**Published:** 2024-02-28

**Authors:** Tsima Abou Kors, Linda Hofmann, Annika Betzler, Kathrina Payer, Martin Bens, Jens Truong, Adrian von Witzleben, Jaya Thomas, Johann M. Kraus, Randa Kalaajieh, Diana Huber, Jasmin Ezić, Julian Benckendorff, Jens Greve, Patrick J. Schuler, Christian H. Ottensmeier, Hans A. Kestler, Thomas K. Hoffmann, Marie-Nicole Theodoraki, Cornelia Brunner, Simon Laban

**Affiliations:** 1 Department of Otorhinolaryngology, Head and Neck Surgery, Ulm University Medical Center, Ulm, Germany.; 2 Fritz Lipmann Institute, Leibniz Institute on Aging, University of Jena, Jena, Germany.; 3 Cancer Sciences Unit, University of Southampton, Faculty of Medicine, Southampton, United Kingdom.; 4 Institute for Medical Systems Biology, Ulm University, Ulm, Germany.; 5 Institute of Pathology, Ulm University Medical Center, Ulm, Germany.; 6 Institute of Systems, Molecular and Integrative Biology, Liverpool Head and Neck Center, University of Liverpool, Faculty of Medicine, Liverpool, United Kingdom.

## Abstract

Patients with oropharyngeal squamous cell carcinoma (OPSCC) caused by human papilloma virus (HPV) exhibit a better prognosis than those with HPV-negative OPSCC. This study investigated the distinct molecular pathways that delineate HPV-negative from HPV-positive OPSCC to identify biologically relevant therapeutic targets. Bulk mRNA from 23 HPV-negative and 39 HPV-positive OPSCC tumors (*n* = 62) was sequenced to uncover the transcriptomic profiles. Differential expression followed by gene set enrichment analysis was performed to outline the top enriched biological process in the HPV-negative compared with HPV-positive entity. *INHBA*, the highest overexpressed gene in the HPV-negative tumor, was knocked down. Functional assays (migration, proliferation, cell death, stemness) were conducted to confirm the target's oncogenic role. Correlation analyses to reveal its impact on the tumor microenvironment were performed. We revealed that epithelial-to-mesenchymal transition (EMT) is the most enriched process in HPV-negative compared with HPV-positive OPSCC, with *INHBA* (inhibin beta A subunit) being the top upregulated gene. *INHBA* knockdown downregulated the expression of EMT transcription factors and attenuated migration, proliferation, stemness, and cell death resistance of OPSCC cells. We uncovered that *INHBA* associates with a pro-tumor microenvironment by negatively correlating with antitumor CD8^+^ T and B cells while positively correlating with pro-tumor M1 macrophages. We identified three miRNAs that are putatively involved in repressing *INHBA* expression. Our results indicate that the upregulation of INHBA is tumor-promoting. We propose INHBA as an attractive therapeutic target for the treatment of *INHBA*-enriched tumors in patients with HPV-negative OPSCC to ameliorate prognosis.

**Significance:**

Patients with HPV-negative OPSCC have a poorer prognosis due to distinct molecular pathways. This study reveals significant transcriptomic differences between HPV-negative and HPV-positive OPSCC, identifying *INHBA* as a key upregulated gene in HPV-negative OPSCC's oncogenic pathways. INHBA is crucial in promoting EMT, cell proliferation, and an immunosuppressive tumor environment, suggesting its potential as a therapeutic target for HPV-negative OPSCC.

## Introduction

Head and neck squamous cell carcinoma (HNSCC) is the sixth most common cancer worldwide, with a high incidence in developing countries. The majority of HNSCCs is caused by tobacco and alcohol abuse, as well as infection with high-risk human papillomavirus (HPV; ref. 1). Oropharyngeal squamous cell carcinoma (OPSCC) is an HNSCC subtype affecting the oropharynx, which includes the tonsils, the base of the tongue, and the back of the throat (2). HPV was identified as a major risk factor for this type of cancer. However, not all cases of OPSCC are caused by HPV (3). HPV has been found to impact the prognosis of OPSCC significantly. Studies have shown that patients with HPV-driven OPSCC have a better prognosis than those with HPV-negative OPSCC (3–6). HPV-positive OPSCC tends to have a more indolent course with slower growth (7). In contrast, HPV-negative OPSCC is associated with a more aggressive clinical course, poorer prognosis, and higher resistance to therapy (7, 8). There is a high medical need to improve outcomes of patients with HPV-negative OPSCC.

Epithelial-to-mesenchymal transition (EMT) is a biological process that allows epithelial cells to acquire a mesenchymal phenotype characterized by increased motility, invasiveness, and resistance to therapy. Studies have shown that the activation of EMT-associated signaling pathways, such as TGFβ, is linked to increased invasiveness and poor prognosis (9). INHBA (inhibin beta A subunit) is a protein belonging to the TGFβ family. Preliminary bioinformatic analysis of public datasets has shown that *INHBA* is overexpressed in various solid tumors, including head and neck cancer, compared with respective healthy tissues (10–16).

Despite the significant progress in understanding the molecular processes underlying OPSCC, the fundamental mechanisms contributing to the aggressiveness of HPV-negative OPSCC are poorly understood, and the available therapeutic targets for this cancer subtype are limited. Current adjuvant treatments for HPV-negative OPSCC, such as radiotherapy, chemotherapy, and immunotherapy, have moderate efficacy in mitigating recurrence and are sometimes associated with significant morbidity and toxicity (17). Therefore, there is a need to identify biologically relevant therapeutic targets for HPV-negative OPSCC, which could, in turn, improve the treatment of this cancer subtype and poor patient outcome. To explore the differentially regulated molecular mechanisms between HPV-negative and HPV-positive OPSCC primary tumors, this study systematically compared the bulk mRNA profiles of the two subentities to outline an appropriate therapeutic target and evaluate its functionally oncogenic role.

## Materials and Methods

### Patient Samples and External Cohort

A total of 62 patients with OPSCC were included in our study cohort (Ulm). A total of 51 representative tissue samples from primary tumor were obtained during surgery at the University Hospital Ulm, Germany, and 11 were provided by C. Ottensmeier from Southampton University, Southampton, UK. The designated ethical committee has given their approval for this study (Ulm University: approval number 222/13; 90/15; UK Medical Research and Ethics Committee: approval number MREC 09/H0501/90). Informed written consent was obtained before patient samples were taken between July 2013 and August 2022. All procedures involving patient sample collection were conducted in full compliance with the ethical standards outlined in the Declaration of Helsinki. HPV status was determined by RNA sequencing (RNA-seq), PCR for HPV DNA, and p16 IHC. The Cancer Genome Atlas (TCGA) gene and miRNA expression values along with clinical parameters of 70 OPSCC samples were downloaded using the R package TCGAbiolinks (2.23.2). TCGA patient with barcode TCGA-BB-A6UM lacks miRNA data. Hence, only 69 samples were included in the miRNA analysis. HPV status of TCGA samples was determined utilizing the metdata provided on p16 IHC and HPV-DNA ISH. Missing HPV status was determined by collecting HPV oncogenes normalized RNA reads per million and applying the cutoff recommended in a previous study (18).

### RNA-seq

The AllPrep DNA/RNA Mini Kit (Qiagen, 80204) was used to extract total RNA from snap-frozen tumor tissues. Quality of total RNA was evaluated using an Agilent 2100 Bioanalyzer Instrument (Agilent RNA 6000 Pico). The next-generation sequencing method from Illumina was used to sequence RNA samples, following the manufacturer's recommendations. Briefly, libraries were made from 500 ng of input material using TruSeq Stranded mRNA. Libraries of 51 samples were pooled and single-end sequenced in one lane of the HiSeq 2500/NovaSeq 6000 System. A total of 11 samples libraries were paired-end sequenced on HiSeq2500 platform. Sequence data were converted to FASTQ format using bcl2fastq (2.20.0.422). After being mapped to the human genome (hg38) using STAR (2.0.9) and multimapping reads being eliminated, high-quality readings were converted to gene-specific read counts for identified genes using featureCounts (2.0.0). As a fraction of all reads, almost 75% of the total reads were uniquely mapped. Unmapped reads were matched to HPV high-risk type genomes using a viGen bioinformatic approach (19). If samples had at least 500 reads for either the HPV E6 or E7 RNA or at least 500 reads for all HPV oncogenes combined (E1, E2, E4, E5, E6, E7, L1, L2), they were considered HPV-positive.

### Cell Culture

The HPV-negative OPSCC cell line UDSCC1 (RRID: CVCL_E324, provided by TKH) and the HPV16-positive hypopharyngeal squamous cell carcinoma cell line UDSCC2 (RRID: CVCL_E325, provided by TKH) were cultured in DMEM (Gibco, 41965-039) supplemented with 10% FBS (Bio&Sell, FBS.S 0615) and 1% MEM non-essential amino acids (Gibco, catalog no.: 11140-035) at 37°C, 5% CO_2_ and >95% relative humidity. The identity of the cell lines was proven in July 2023 by short tandem repeats analysis. Cultures were checked monthly for absence of *Mycoplasma* contamination (VenorGeM Advance, Minerva Biolabs GmbH, catalog no.: 11-7024). Experiments were performed on cells until passage 20 after thawing.

### Transfection of UDSCC1 Cells

A total of 250,000 UDSCC1 cells in 2.5 mL complete medium were seeded per well of a 6-well plate (Sarstedt, 83.3920.300) and cultured for 24 hours. After 24 hours, cells were transfected with *INHBA* Silencer predesigned siRNA (Thermo Fisher Scientific, AM16708, assay ID siRNA1: 11093 and assay ID siRNA2: 144964) or Silencer Select Negative Control #2 siRNA (Thermo Fisher Scientific, 4390846). For each well, 75 pmol of siRNA were mixed with reduced serum medium (Opti-MEM, Gibco, catalog no.: 31985-047) and 4 µL Lipofectamine RNAiMAX (Thermo Fisher Scientific, 13778-030). The mix was incubated at room temperature for 15 minutes and was added dropwise to each well. After transfection, cells were cultured for another 24 hours (t24) or 48 hours (t48) at which functional assays were conducted, as indicated in the figure legends.

### qRT-PCR

Cells were harvested using 350 µL RLT buffer from the RNeasy Mini Kit (Qiagen, 74104) supplemented with 2-mercaptoethanol (1:100). RNA was isolated according to manufacturer's instruction and RNA concentrations were determined using a TECAN spectrophotometer. A total of 1,000 ng RNA were reverse transcribed using QuantiTect Reverse Transcription Kit (Qiagen, 205313) and qRT-PCR was subsequently performed using the QuantiNova SYBR Green PCR Kit (Qiagen, 208056), both according to manufacturer's instruction. qRT-PCR was run in a Roche Light Cycler 96 using the following program: 2 minutes of initial heat activation at 95°C, followed by two-step cycling of 5 seconds denaturation at 95°C and 10 seconds combined annealing and extension at 60°C, repeated for 40 cycles. Following primers were manufactured by Biomers: *RPL30* forward 5′-tggtggctgcaaagaagac-3′, *RPL30* reverse 5′-gcagttgttagcgagaatgac-3′, *INHBA* forward 5′-aagtcggggagaacgggtatgtgg-3′, *INHBA* reverse 5′-tcttcctggctgttcctgactcg-3′, *SNAI2* forward 5′-cgaactggacacacatacagtg-3′, *SNAI2* reverse 5′-ctgaggatctctggttgtggt-3′, *SNAI1* forward 5′-tcggaagcctaactacagcga-3′, *SNIA1* reverse 5′-agatgagcattggcagcgag-3′, *TWIST1* forward 5′-ggagtccgcagtcttacgag-3′, *TWIST1* reverse 5′-tctggaggacctggtagagg-3′. Experiments were performed in duplicate and with negative controls. Ribosomal Protein L30 (*RPL30*) was used as the normalization control. The delta Ct value (ΔCt) was calculated between the target and the *RPL30* mean of the same condition. The ΔΔCt value was calculated between *INHBA* knockdown (KD) and mean Mock KD (considered as 1).

### Western Blot Analysis

Cell lysis was performed using RIPA buffer supplemented with protease and phosphatase inhibitors (1 tablet/10 ml buffer) (Roche, 04906837001, 04693159001) for 30 minutes on ice. Cell lysates were centrifuged at 12,000 × *g*, 30 minutes, 4°C and protein content was determined using Pierce bicinchoninic acid Protein Assay (Thermo Fisher Scientific, 23225). A total of 20 µg proteins were mixed with 4x Laemmli Buffer (Bio-Rad, 1610747) supplemented with 2-Mercaptoethanol and denatured at 95°C for 5 minutes. Samples and 7 µL of protein ladder (Bio-Rad, 161-0374) were separated on 12% Mini-PROTEAN Precast Gels (Bio-Rad, 120 V) and were transferred to polyvinylidene difluoride membranes using Trans-Blot Turbo Transfer Kit (Bio-Rad, 1704272). Membranes were blocked with 5% BSA/Tris-buffered saline with Tween 20 (TBS-T) overnight at 4°C and incubated with anti-INHBA antibody (Abcam, ab128958, RRID:AB_11144514, 1: 5,000 in 5% BSA/TBS-T), anti-E-Cadherin antibody (BD, 610182, RRID:AB_397581, 1:1,000 in 5% milk/TBS-T) and anti-GAPDH antibody (Santa Cruz Biotechnology, sc-25778, RRID: AB_10167668, 1:2,000 in 5% milk/TBS-T) overnight at 4°C. Then, membranes were washed 3x with TBS-T and incubated with goat anti-rabbit IgG secondary antibody, HRP (Thermo Fisher Scientific, 31460, RRID: AB_228341 1:10,000 in 5% BSA/TBS-T) for 40 minutes at room temperature. Membranes were washed 4x with TBS-T and developed using enhanced chemiluminescence Substrate (Thermo Fisher Scientific, 34076) and ChemiDoc Imaging System (Bio-Rad). Blots were analyzed using Image Lab 6.0.1 software (Bio-Rad).

### Scratch Assay

The cell monolayer was scratched at the day of transfection using a pipette tip and documented every 24 hours using an Olympus CK30 microscope (Zeiss) at 10-fold magnification. Image processing was performed using python (3.8.5). scikit-image (0.18.3) was used to read images and the entropy function was used to calculate entropy at each pixel position. Images were plotted using matplotlib (3.5.0) and Otsu method was utilized to compute the optimal threshold for image binarization. Area in pixels (*A*) with entropy lower than the threshold was calculated using numpy (1.21.2) and used as a proxy for the open wound area. Open wound percentage was calculated as follows, where *t* denotes any timepoint and 0 denotes the starting timepoint:



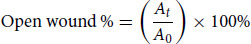



### Proliferation Assay

Immediately before transfection, cells were stained with 2.5 µmol/L CellTrace CFSE (Thermo Fisher Scientific, C34554) at 37°C for 20 minutes in the dark, according to manufacturer's instruction for adherent cells. Cells were harvested using Trypsin/Ethylenediaminetetracetic acid (EDTA) (PAN-Biotech, P10-023100) and proliferation was measured on a Gallios flow cytometer (Beckman Coulter).

### Cell Death Assay

Apoptosis and necrosis were measured using FITC Annexin V Apoptosis Detection Kit with 7-Aminoactinomycin D (7-AAD) (BioLegend, 640922) according to manufacturer's instruction. Briefly, cells were washed with annexin V binding buffer, provided with the kit, and resuspend in 100 µL annexin V binding buffer. Cells were stained with 5 µL FITC annexin V and 5 µL 7-AAD for 15 minutes in the dark and measured on a Gallios flow cytometer (Beckman Coulter).

### Colony Formation Assay

A total of 1,000 cells per well were seeded in 6-well plates, transfected the next day and cultured for 2 weeks without medium change. Then, cells were washed with ice-cold PBS (Gibco, 14190-094) and fixed with prechilled methanol (Thermo Fisher Scientific, 10365710) for 15 minutes before being rinsed once more with PBS. Colonies were stained with 0.5% crystal violet dye (Carl Roth, T123.1). Images were taken using Keyence BZ-X microscope (Keyence) at 5-fold magnification to scan the entire well. Image processing was performed using BZ-II Analyzer 1.0 (Keyence).

### Aldehyde Dehydrogenase Activity Assay

Aldehyde dehydrogenase (ALDH) activity assay (Abcam, ab155893) and data analysis were performed following the manufacturer's instructions. Absorption was measured using TECAN spectrophotometer at 450 nm.

### Data Analysis and Statistical Analysis

Data analysis was performed in R (4.1.1). Data wrangling was carried out utilizing tidyverse (1.3.1), data.table (1.14.2), and dplyr (1.0.9). Differential expression analysis was performed using deseq2 (1.34.0), and the shrinkage estimator used was “apeglm” (20). The threshold for differentially expressed genes was determined with an absolute log fold change (LFC) >1 and a FDR < 0.05 with a correction for batch effect. The RNA expression transcript per million (TPM) values were used for correlation and survival analysis binarization. Correlation matrices were generated using corrplot (0.92). The Spearman correlation was calculated using R base stats package. Correlations with *P* value < 0.05 were considered significant. Hallmark gene sets were loaded using msigdbr (7.5.1). Gene set enrichment analysis (GSEA) with 1,000 permutations was performed and plotted using fgsea (1.20.0); gene sets with FDR < 0.05 were considered significantly regulated. Binarization of patients based on expression cutoffs was done using matrixStats package (0.61.0). Survival analysis was conducted by fitting survival curves using the survival package (3.3-1) and generating Kaplan–Meier plots utilizing the survminer package (0.4.9). The restricted mean survival time (RMST) was computed and plotted using the survRM2 package (1.0-4). The immunedeconv (2.0.4) R wrapper package was used to perform immune deconvolution on RNA-seq data using the quanTIseq program as method to predict M1 and M2 macrophages, monocytes, CD4^+^ regulatory and non-regulatory T cells, CD8^+^ T cells, B cells, neutrophils, natural killer cells, and myeloid dendritic cells infiltration ratios. Venn diagrams were generated using ggvenn (0.1.9). Assessment of complementarity between miRNA and 3′-UTR (untranslated region) of INHBA and targeted prediction was done utilizing miRDB (21) and TargetScan 8.0 (22). Visualization was done using ggplot2 (3.3.6). Clinical data summary was generated using summarytools (1.0.1).

### Data Availability Statement

The data generated in this study are publicly available in Sequence Read Archive database at PRJNA967751.

## Results

### Study Population

The clinicopathologic characteristics of the Ulm and TCGA OPSCC cases (*n* = 62; *n* = 70) are described in Table 1. There were no significant statistical differences between the cohorts.

**TABLE 1:** Clinicopathologic features of patients with OPSCC in Ulm and TCGA cohorts

	Ulm Cohort (*n* = 62)		TCGA Cohort (*n* = 70)		
	Age (years) Range: 38–79 Mean (SD): 60.5 (9.1)		Age (years) Range: 35–79 Mean (SD): 56.3 (9.4)	
Characteristics	*n*	%	*n*	%	*P*-value
Gender					
Male	50	80.6	62	88.6	
Female	12	19.4	8	11.4	0.23
Missing	0	0	0	0	
Tumor stage					
T1	7	11.3	12	17.2	
T2	28	45.2	28	40	
T3	19	30.6	19	27.1	0.49
T4	8	12.9	8	11.4	
Missing	0	0	3	4.3	
Nodal status					
N0	11	19.3	20	28.6	
N+	51	80.7	47	67.1	0.07
Missing	0	0	3	4.3	
Distant metastasis					
M0	62	100	64	91.4	
M1	0	0	1	1.4	0.06
Missing	0	0	5	7.2	
HPV					
Positive	39	62.9	45	64.3	
Negative	23	37.1	25	35.7	1
Missing	0	0	0	0	
Vital status					
Dead	13	21.0	13	18.6	
Alive	49	79.0	57	81.4	0.83
Missing	0	0	0	0	

### GSEA Identified *INHBA* as an EMT driver in HPV-negative Tumors

**FIGURE 1:**
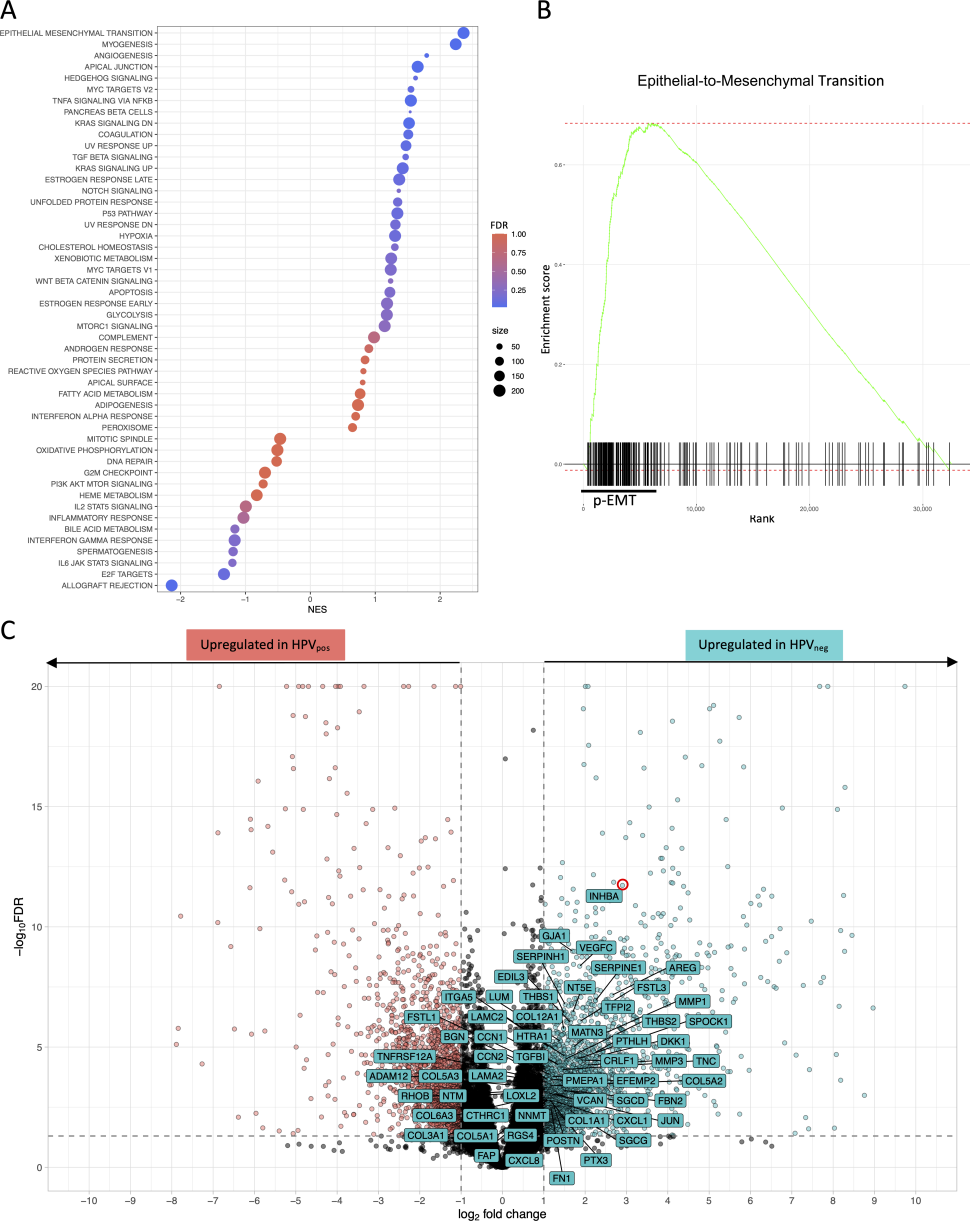
GSEA identifying *INHBA* as a leading driver of EMT in HPV-negative tumors. **A,** Dot plot of pathways enriched and depleted in HPV-negative compared with HPV-positive OPSCC tumor as determined by GSEA of transcriptomes from *n* = 62 primary tumor samples (Ulm cohort). Results were ranked according to the NES, dots were colored according to FDR and size was determined by the number of genes. **B,** GSEA plot of the top pathway (EMT) showing enrichment score (green curve), position of genes (black lines) with the leading edge (p-EMT), NES, and FDR. **C,** Volcano plot showing the log_2_ fold change of genes upregulated in HPV-positive (red) and HPV-negative OPSCC (blue), with *INHBA* (red circle) as the highest upregulated leading edge in HPV-negative tumors.

**FIGURE 2:**
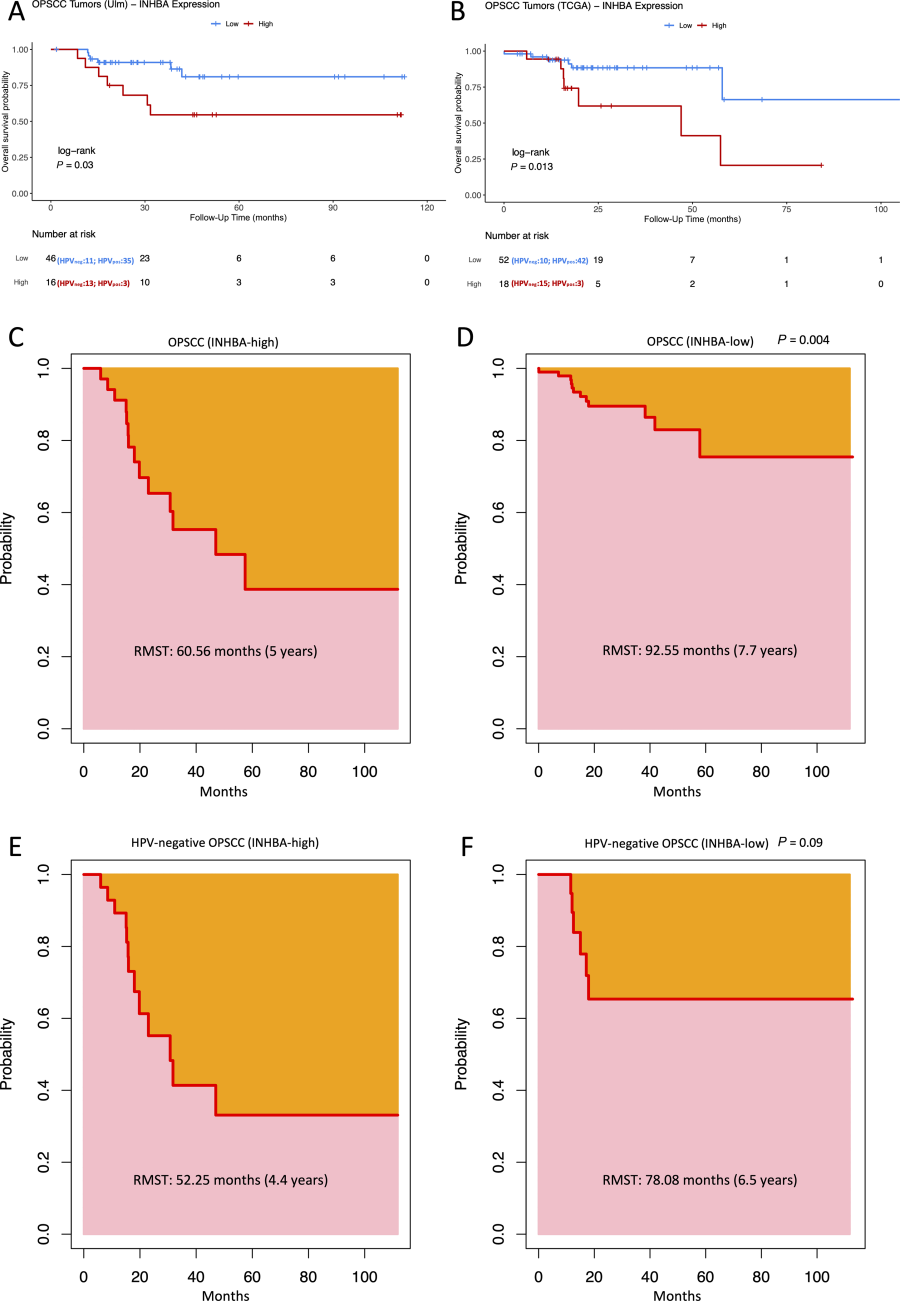
Kaplan–Meier survival analysis and RMST in patients with OPSCC based on *INHBA* expression levels. **A** and **B,** Kaplan–Meier plots with log-rank test for overall survival of patients with OPSCC (**A:** Ulm cohort, **B:** TCGA cohort) based on tumor INHBA expression. Blue indicates low and red indicates high *INHBA* expression, as determined using Q3 as cutoff. (**C–F**) RMST defined as the area under the survival curve (pink) based on *INHBA* expression status (**C:***INHBA*-high in all tumors, **D:***INHBA*-low in all tumors, **E:***INHBA*-high in HPV-negative tumors, **F:***INHBA*-low in HPV-negative tumors).

To investigate the differential regulation of biological processes underlying the differences between HPV-negative and HPV-positive OPSCC, we performed differential expression analysis of 23 HPV-negative and 39 HPV-positive tumors followed by GSEA. The analysis revealed significant (FDR < 0.05) enrichment for gene sets related to EMT [normalized enrichment score (NES) = 2.36], myogenesis (NES = 2.24), angiogenesis (NES = 1.79), components of the apical junction complex (NES = 1.65), TNFα signaling via NFκB (NES = 1.55), and downregulated genes by KRAS activation (NES = 1.52), and attenuation of upregulated genes during transplant rejection (NES = −2.14) in HPV-negative versus HPV-positive tumors (Fig. 1A; Supplementary table S1). EMT was the top enriched biological process in the HPV-negative tumor (Fig. 1B). The EMT gene set included 200 genes, with 127 identified as the leading edge of enrichment (p-EMT). Fifty-five of the p-EMT were shown to be significantly upregulated in the HPV-negative tumor (FDR < 0.05). *INHBA* exhibited the highest upregulation with an LFC of 2.9 (Fig. 1C; Supplementary Table S2). The impact of *INHBA* expression on the clinical outcome of patients with OPSCC was evaluated (Ulm cohort) and validated (TCGA cohort) by survival analysis with the log-rank test. Patients were binarized into high and low expression categories based on the third quantile (Q3) of TPM as a cutoff. Most patients in the high expression category were HPV-negative (Ulm: 81%; TCGA: 83%), which mirrors the enrichment analysis results. Relative expression levels of *INHBA* significantly impacted the overall survival of patients. Patients with higher expression levels had a worse prognosis than those with low levels of *INHBA* (Fig. 2A and B). When evaluated by the *INHBA* expression status of the two cohorts, the RMST recorded was lower by 31.99 months in *INHBA*-high versus *INHBA*-low OPSCC (60.56 vs. 92.55, respectively; Fig. 2C and D). In HPV-negative patients, the RMST recorded was lower by 25.83 months in *INHBA*-high versus *INHBA*-low OPSCC (52.25 vs. 78.08, respectively, Fig. 2E and F) but did not reach statistical significance (*P* = 0.09). Our data outlines *INHBA* as an HPV-dependent prognostic biomarker in OPSCC by delineating a subpopulation of patients with HPV-negative OPSCC, overexpressing *INHBA* in the primary tumor.

### *INHBA* Regulates the Expression of the EMT Transcription Factors and Enhances Migration in an HPV-negative OPSCC Cell Line

**FIGURE 3:**
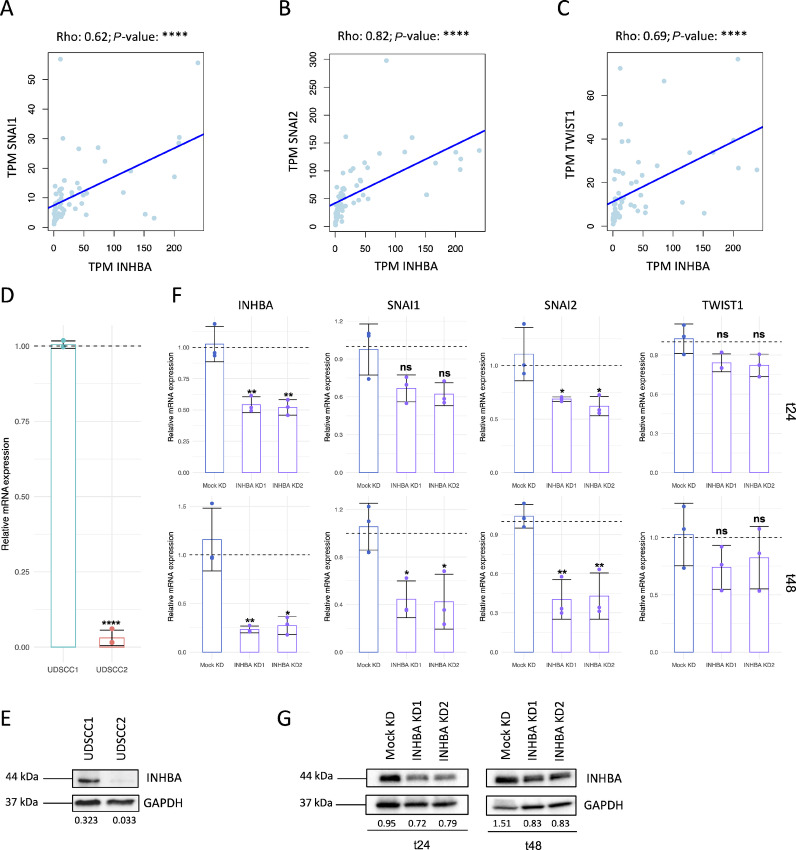
*INHBA* expression in OPSCC tumor and cell lines and its correlation with EMT transcription factors. Correlation of *INHBA* expression with the expression of *SNAI1* (**A**), *SNAI2* (**B**), and *TWIST1* (**C**) in OPSCC tumors (Ulm cohort). TPM = transcripts per million. **D,** Relative expression of *INHBA* mRNA in native UDSCC1 (HPV-negative) and UDSCC2 (HPV-positive) cells as determined by qRT-PCR and normalized to RPL30 and UDSCC1. Results are shown as mean ± SD of *n* = 6 replicates. *INHBA* expression was not detected in *n* = 3 UDSCC2 samples. Expression level between UDSCC1 and UDSCC2 was compared by unpaired *t* test with **, *P* ≤ 0.01. **E,** Representative Western blot analysis (of *n* = 3 replicates) showing *INHBA* protein expression in native UDSCC1 and UDSCC2 cells. Numbers below blots indicate relative INHBA intensity normalized to GAPDH. **F,** Relative expression of *INHBA*, *SNAI1*, *SNAI2*, and *TWIST1* mRNA in UDSCC1 cells upon transfection with Mock siRNA [Mock knockdown (KD)] and two *INHBA* siRNAs (*INHBA* KD1 and KD2) after 24 hours (t24) and 48 hours (t48). Target gene expression was determined by qRT-PCR and normalized to RPL30 and Mock KD. Results are shown as mean ± SD of *n* = 3 replicates. Expression level between Mock KD and *INHBA* KD1/2 was compared by unpaired *t* test with *, *P* ≤ 0.05; **, *P* ≤ 0.01; and ns, *P* > 0.05. **G,** Representative Western blot analysis (of *n* = 3 replicates) showing INHBA protein expression in Mock KD and *INHBA* KD1/KD2 at t24 and t48. Numbers below blots indicate relative INHBA intensity normalized to GAPDH.

**FIGURE 4:**
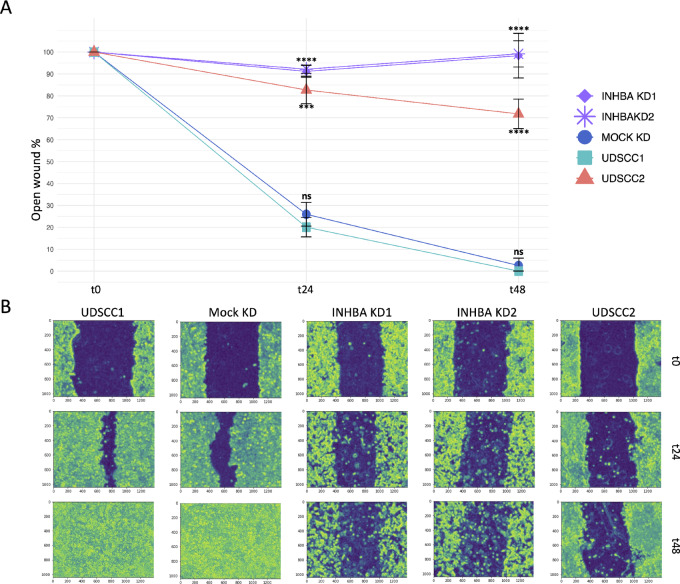
Cell migration upon INHBA knockdown. **A,** Migration of native UDSCC1 cells, Mock KD, *INHBA* KD1/KD2 and native UDSCC2 cells as assessed by scratch assay. Results are shown as mean ± SD of the open wound area (*n* = 3) at baseline (t0), t24, and t48. Conditions were compared with native UDSCC1 using unpaired *t* test with ***, *P* ≤ 0.001; ****, *P* ≤ 0.0001; and ns, *P* > 0.05. **B,** Representative images of processed light microscopy pictures taken at 10-fold magnification of each condition at t0, t24, and t48.

A strong positive correlation was detected between the expression of *INHBA* and EMT transcription factors *SNAI1* (Rho = 0.62), *SNAI2* (Rho = 0.82), and *TWIST1* (Rho = 0.69) in the primary tumor (Fig. 3A–C). INHBA was found to be differentially expressed under native conditions in UDSCC1 (HPV-negative) and UDSCC2 (HPV-positive) cell lines, with significantly higher mRNA and protein levels in UDSCC1 (Fig. 3D and E). To verify the role of INHBA in regulating the expression of EMT transcription factors in HPV-negative tumors, two siRNAs against *INHBA* (KD1 and KD2) were transfected into UDSCC1, and UDSCC2 was used as a positive control to evaluate whether the knockdown of *INHBA* relatively shifts the oncogenic features of UDSCC1 to a phenotype comparable to that of UDSCC2. *INHBA* knockdown efficiencies were evaluated on RNA level (Fig. 3F, KD1: 47% after 24 hours, 80% after 48 hours, KD2: 49% after 24 hours, 76% after 48 hours) and validated on protein level (Fig. 3G, KD1: 24% after 24 hours, 45% after 48 hours, KD2: 17% after 24 hours, 45% after 48 hours). *INHBA* knockdown significantly downregulated the expression level of *SNAI2* 24 and 48 hours after transfection and *SNAI1* 48 hours after transfection, while *TWIST1* showed a nonsignificant decrease (Fig. 3F). *INHBA* knockdown showed a modest upregulation of E-Cadherin protein levels (Supplementary Fig. S1). As EMT enhances the migratory ability of cells, we examined the *INHBA* knockdown effect on the migratory potential of UDSCC1. The wound healing assay revealed that *INHBA* knockdown decreased the wound closure capability of UDSCC1 cells and rendered them less motile compared with the negative control and even UDSCC2 cells (Fig. 4A and B). These findings indicate that *INHBA* initiates EMT in HPV-negative OPSCC cells by primarily upregulating the expression of *SNAI2*, resulting in the promotion of cancer cell motility.

### *INHBA* Potentiates Proliferation and Attenuates Cell Death of UDSCC1

**FIGURE 5:**
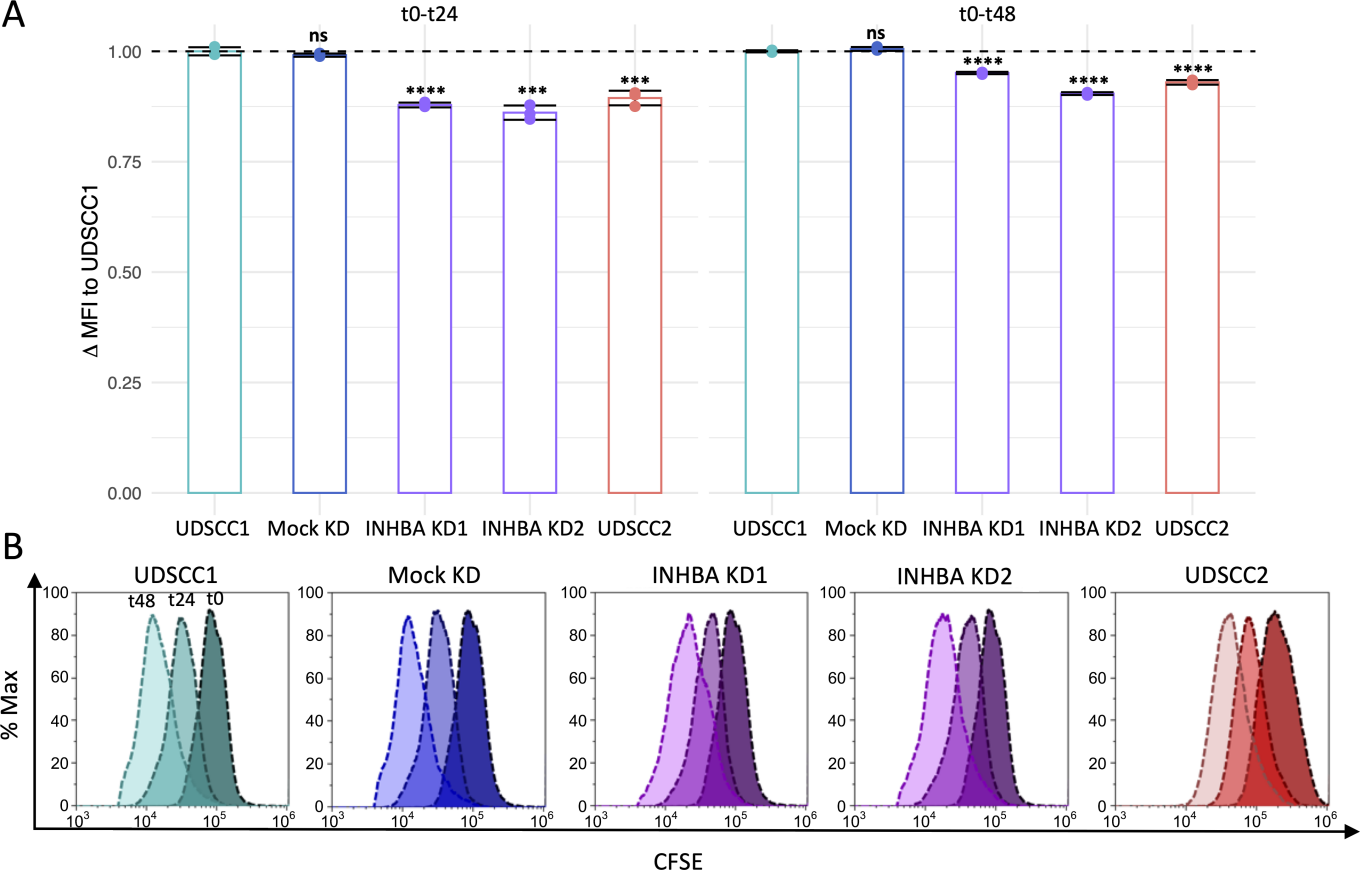
Cell proliferation upon *INHBA* knockdown. **A,** Proliferation of native UDSCC1 cells, Mock KD, *INHBA* KD1/KD2 and native UDSCC2 cells as assessed by carboxyfluorescein succinimidyl ester (CFSE) assay and flow cytometry. Results are shown as mean ± SD of the ΔMFI (mean fluorescence intensity) normalized to UDSCC1 between t0 and t24, or t0 and t48 (*n* = 3). Conditions were compared with native UDSCC1 using unpaired *t* test with ***, *P* ≤ 0.001; ****, *P* ≤ 0.0001; and ns, *P* > 0.05. **B,** Representative flow cytometry histograms for each condition with the right peak representing t0, central peak representing t24 and left peak representing t48.

**FIGURE 6:**
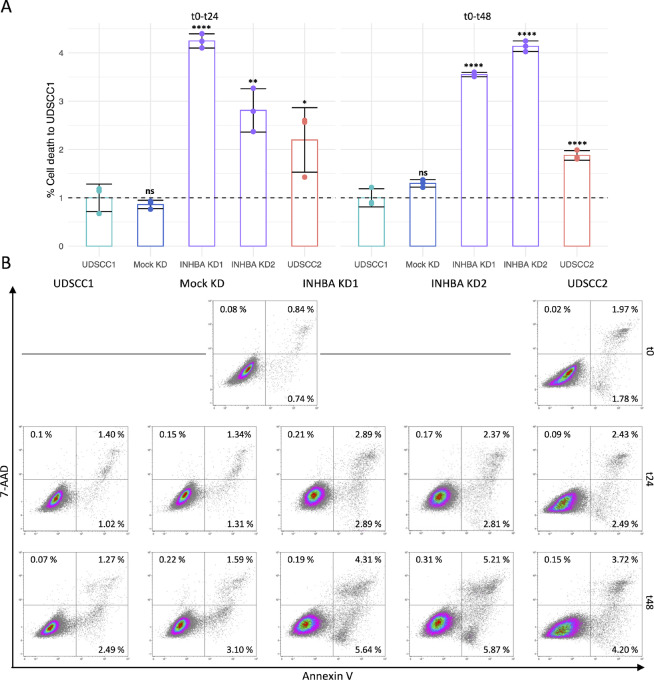
Cell death upon *INHBA* knockdown. **A,** Cell death of native UDSCC1, Mock KD, *INHBA* KD1/KD2 and native UDSCC2 cells as assessed by Annexin V/7-AAD staining and flow cytometry. Results are shown as mean ± SD of the percentages of dead cells normalized to UDSCC1 (*n* = 3). Conditions were compared with native UDSCC1 using unpaired *t* test with *, *P* ≤ 0.05; **, *P* ≤ 0.01; ****, *P* ≤ 0.0001; and ns, *P* > 0.05. **B,** Representative flow cytometry plots for each condition. Gates show early apoptotic in the lower right quadrant, late apoptotic in the upper right quadrant and necrotic cell populations in the upper left quadrant. t0-plots represent the baseline timepoint right before transfection.

To further elucidate the role of *INHBA* in promoting the aggressiveness of HPV-negative OPSCC cells, we examined its knockdown effect on the proliferation capacity and cell death resistance in UDSCC1. *INHBA* knockdown notably reduced the proliferative ability of UDSCC1 cells rendering it comparable to that of the less proliferative UDSCC2 (Fig. 5A and B). In addition, the knockdown of *INHBA* significantly promoted the death of UDSCC1 cells, yielding a higher level of primarily late apoptotic but also early apoptotic and necrotic cell percentages (Fig. 6A and B). These results indicate that *INHBA* overexpression in HPV-negative tumors promotes the aggressiveness of cancer cells by enhancing proliferation while attenuating cell death simultaneously.

### *INHBA* Associates with the Expression of HNSCC Stemness-related Markers and Potentiates Clonal Expansion

**FIGURE 7:**
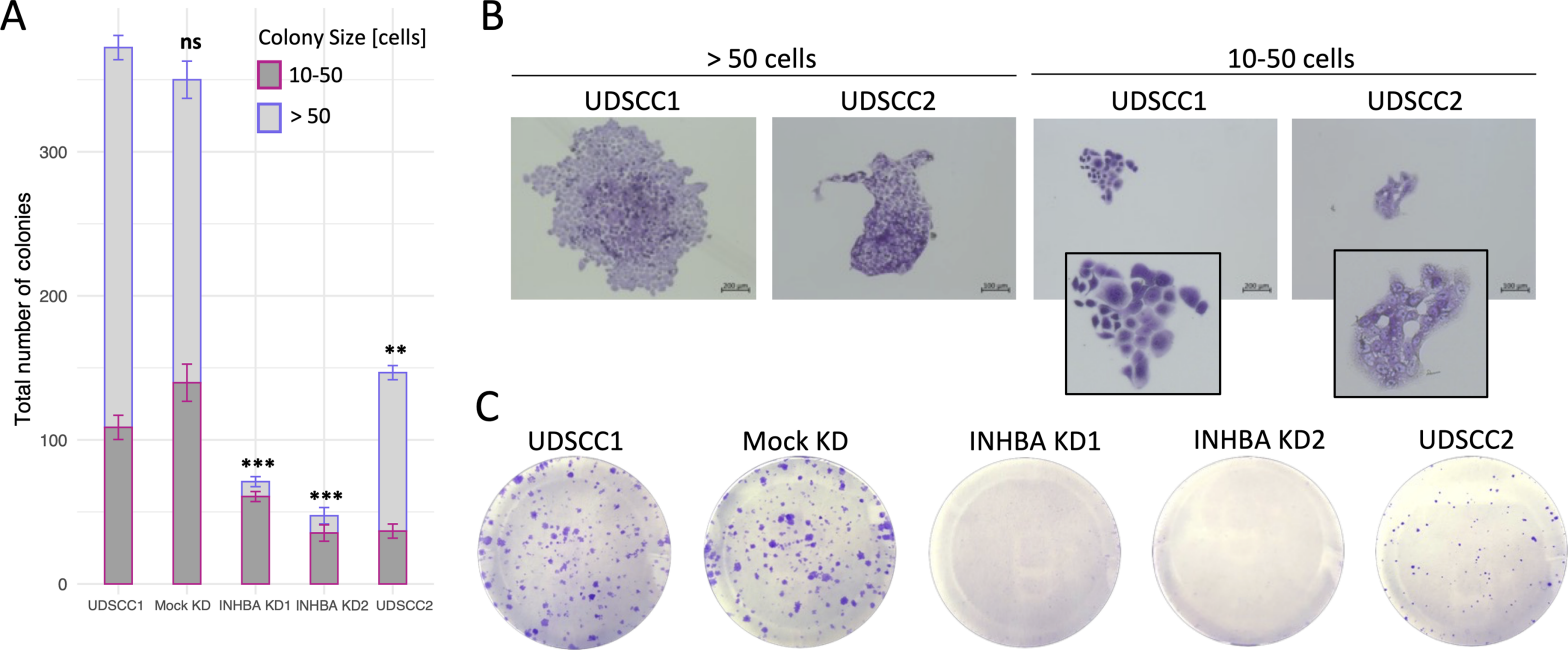
Colony formation ability upon *INHBA* knockdown. **A,** Ability for clonal expansion of native UDSCC1 cells, Mock KD, *INHBA* KD1/KD2, and UDSCC2 cells as assessed by colony formation assay. Colonies between 10 and 50 cells were considered small (red) and colonies greater 50 cells were considered big (blue). Results are shown as mean ± SEM of the number of total colonies (*n* = 3). Conditions were compared with native UDSCC1 using unpaired *t* test with **, *P* ≤ 0.01; ***, *P* ≤ 0.001; and ns, *P* > 0.05. **B,** Representative images of small and big colonies in UDSCC1 and UDSCC2 cells stained with crystal violet. **C,** Representative images of colony formation for each condition.

**FIGURE 8:**
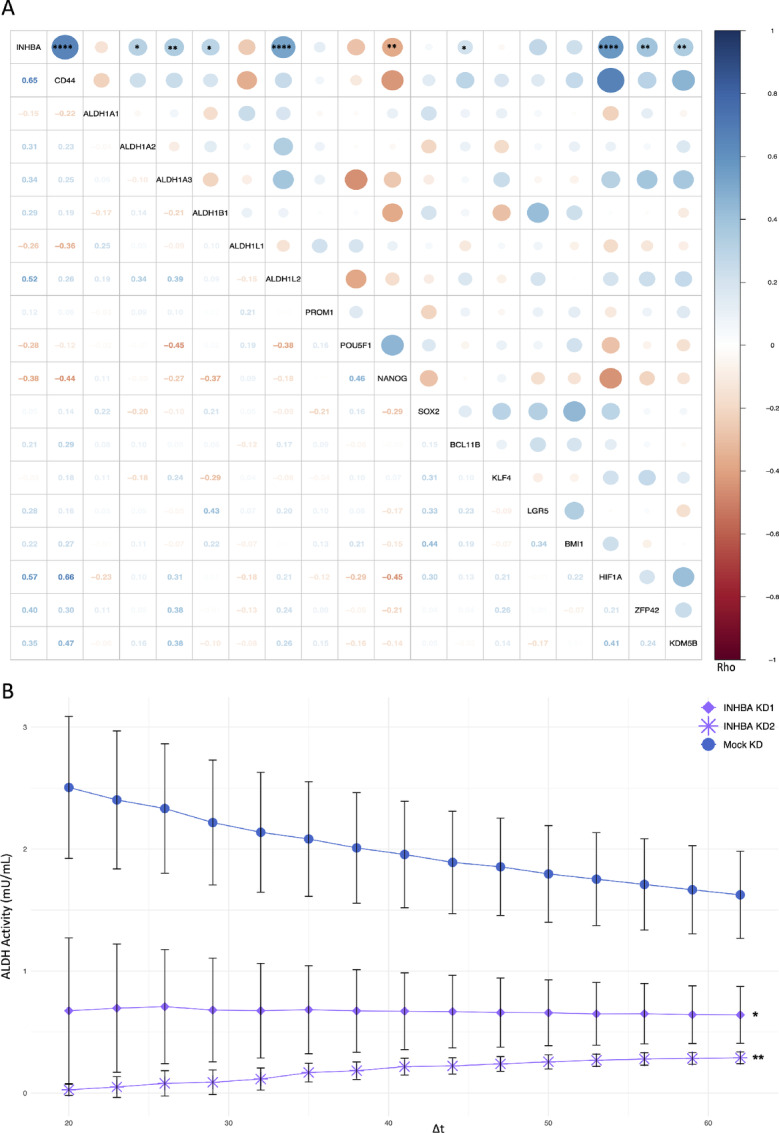
*INHBA* correlates with stemness attributes. **A,** Correlation matrix between *INHBA* and stemness-related markers showing Spearman rho in scaled dots and *P*-value symbols (*, *P* ≤ 0.05; **, *P* ≤ 0.01; ****, *P* ≤ 0.0001) in the upper triangular matrix and Spearman rho values in the lower triangular matrix. **B,** Time series of ALDH enzyme activity for Mock KD and INHBA KD1/KD2. Results are shown as mean ± SD of ALDH activity (mU/mL; *n* = 3) and Δt (minutes). *INHBA* KD1/KD2 were compared with Mock KD using unpaired *t* test with *, *P* ≤ 0.05 and **, *P* ≤ 0.01.

As undifferentiated cancer cells, most likely cancer stem cells, are the driving force of clonal expansion, a colony formation assay was performed to investigate whether *INHBA* knockdown influences the ability of single cells, particularly cancer stem cells, to resist the absence of cell-to-cell contact and develop into a colony via clonal expansion. Because this assay aimed not to assess the proliferative potential but rather the ability of single cells to survive and expand, and because INHBA was proven to influence proliferation, small colonies were also counted while keeping the time variable fixed for all conditions. Colonies ranging in size between 10 and 50 cells and those of 50 or more were counted (Fig. 7A and B). Small colonies were relatively more prevalent in UDSCC1 with *INHBA* knockdown. The number of colonies recorded for UDSCC2 and UDSCC1 cells upon *INHBA* knockdown was significantly lower than the negative control cells (Fig. 7A and C). Consequently, expression correlation analysis was done between *INHBA* and previously described HNSCC stemness-related markers in primary tumors. The analysis revealed a significant positive correlation between *INHBA* expression level and nine markers. A positive correlation (Rho > 0.4) was recorded between *INHBA* and *CD44*, *ALDH1L2*, *HIF1A*, and *ZFP42*, simultaneously (Fig. 8A). To functionally validate the association, we performed ALDH enzyme activity assay, as increased ALDH activity serves as a cancer stem cell marker. ALDH activity was significantly reduced in UDSCC1 cells upon *INHBA* knockdown compared with the negative control (Fig. 8B). Overall, these results suggest that INHBA potentially promotes OPSCC stemness.

### *INHBA* Gene Expression Correlates with the Ratio of OPSCC Infiltrating Immune Cells

**FIGURE 9:**
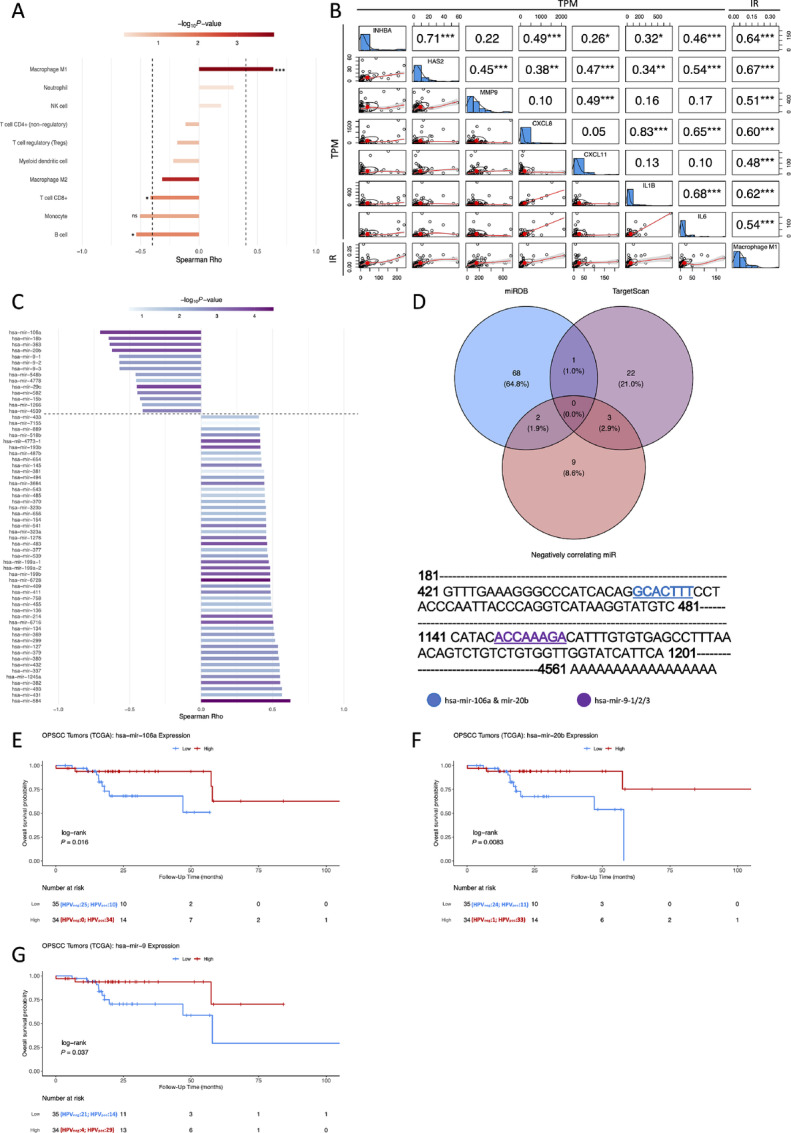
Correlation analyses between *INHBA* expression, immune infiltrates and miRNAs. **A,** Waterfall plot showing Spearman correlation between *INHBA* expression level and infiltrating ratios (IR) of different immune cells, as computed by immune cell deconvolution. Rho > 0.4 was considered relevant (dashed line). *, *P* ≤ 0.05; ***, *P* ≤ 0.001; ns, not significant. **B,** Correlation matrix between *INHBA* and the pro-tumor M1 signature showing Spearman rho and *P*-value symbols (*, *P* ≤ 0.05; **, *P* ≤ 0.01; ***, *P* ≤ 0.001) in the upper triangular matrix and scatter plots in the lower triangular matrix. TPM = transcripts per million. **C,** Waterfall plot showing Spearman correlation between *INHBA* expression level and miRNAs (TCGA cohort). miRNAs with Rho < −0.4 and *P* < 0.05 were considered relevant (dashed line). **D,** Venn diagram showing intersections between negatively correlating miRNAs from C and miRNAs predicted to interact with the 3′UTR of *INHBA* by miRDB and Targetscan resulting in three candidates of interest (hsa-mir-106a, hsa-mir-20b, hsa-mir-9-1/2/3). Their putative binding sites to the *INHBA* 3′UTR are highlighted. **E–G,** Kaplan–Meier plots with log-rank test for overall survival of patients with OPSCC (TCGA cohort) based on tumor miRNA expression of the outlined candidates (**E:** hsa-mir-106a, F: hsa-mir-20b, **G:** hsa-mir-9). Blue indicates low and red indicates high miRNA expression, as determined using median of raw counts as cutoff.

The HNSCC tumor microenvironment is known to be immunosuppressive, particularly in aggressive primary tumors. Hence, we investigated the correlation between *INHBA* expression level and the computed infiltrating ratio of different types of immune cells. The expression level of *INHBA* in primary tumors negatively correlated with the infiltration ratio of CD8^+^ cytotoxic T cells (Rho = −0.42), indispensable in orchestrating the antitumor immune response. Furthermore, a negative correlation (Rho = −0.54) with the level of B-cell infiltrates was revealed. Incidentally, a weak negative correlation (Rho = −0.32) and a strong positive correlation (Rho = 0.64) were disclosed between *INHBA* expression level and M2 and M1 macrophage infiltration level, respectively (Fig. 9A). M1 macrophages are typically known to be critical players in initiating antitumor immunity. Recently, however, a pro-tumor M1 macrophage subtype was described in melanoma (23) and characterized by increasing the formation of a dense matrix around cancer cells via upregulating HA synthase 2 (*HAS2*) due to the activation of the TNFα signaling via NFκB, which in turn, was shown in the current study also to be augmented in HPV-negative OPSCC tumors (Fig. 1A). Hence, we correlated the expression level of the tumor-upregulated gene signature, comprising *HAS2*, *MMP9*, *CXCL8*, *CXCL11*, *IL1B*, and *IL6*, with the ratio of infiltrating M1 macrophages. The genes’ RNA levels positively correlated with M1 ratios infiltrating the OPSCC primary tumor (Fig. 9B). These results suggest that INHBA is a potential immune response modulator by associating with a pro-tumor microenvironment in OPSCC tumors.

### Three miRNAs are Outlined as Putative Regulators of *INHBA* Gene Expression

It is well known that interaction between miRNA and mRNA is a vital mode of gene expression regulation in cancer. We applied a three-step approach to evaluate a possible regulation of *INHBA* expression by miRNAs in the primary tumor. First, we performed an expression correlation utilizing the paired TCGA samples to outline miRNAs that negatively correlate with the *INHBA* mRNA level. Then, we utilized complementarity and interaction prediction of two online databases to delineate the putative interacting candidates. Finally, we assessed the prognostic impact of the outlined miRNAs. A total of 12 miRNAs were found to negatively correlate with *INHBA* expression (Rho < −0.4; *P* < 0.05; Fig. 9C; Supplementary Table S3). A total of 71 and 26 miRNAs were predicted, by miRDB and TargetScan, respectively, to interact with *INHBA* mRNA 3′UTR. Only three of the latter intersected with the negatively correlating miRNA; miR-106a, miR-20b, and miR-9-1/2/3 were the candidates of interest, and their respective binding sites in the 3′UTR were defined (Fig. 9D). After binarizing patients into high and low expression categories based on the median of raw reads as a cutoff, survival analysis showed that most to almost all HPV-negative patients were in the low expression category of the three different miRNAs, thus yielding an inverse prognostic signature to that of *INHBA*. Patients with the lower expression level of all three miRNAs had a worse prognosis (Fig. 9E–G). These findings suggest that miR-106a, miR-20b, and miR-9-1/2/3 likely downregulate the expression of *INHBA*, hindering its oncogenic role in the primary tumor.

## Discussion

Many studies have shown that patients with HPV-positive OPSCC have a better prognosis than HPV-negative patients, mainly due to higher immunogenicity, increased responsiveness to radiation and chemotherapy resulting in lower recurrence rates and distant metastasis (3–8). However, research utilizing unbiased data-driven comparative analysis to delineate relevant therapeutic targets potentially promoting OPSCC aggressiveness in HPV-negative patients is still lacking. In the current study, we applied an exploratory data analysis approach to examine the differentially regulated molecular processes between HPV-negative and HPV-positive OPSCC tumors and to uncover key players contributing to the differential prognosis. We identified EMT as the top enriched process in HPV-negative tumors, with *INHBA* being the leading upregulated gene. The knockdown of *INHBA* in an HPV-negative OPSCC cell line confirmed its functional role in invasion, proliferation, apoptosis resistance, and stemness. We found that *INHBA* is likely involved in accompanying a pro-tumor microenvironment. Moreover, we outlined three miRNAs potentially involved in repressing *INHBA* expression that are downregulated in HPV-negative compared with HPV-positive tumors. These findings reveal the functional role of *INHBA* in OPSCC progression and aggressiveness, suggesting it to be used as potential therapeutic target for *INHBA*-enriched tumors, principally in favor of patients with HPV-negative OPSCC.

*INHBA* is overexpressed in many solid tumors, including HNSCC (10–16). In OPSCC, our study uncovered that the prognostic impact of *INHBA* expression is HPV dependent, with most patients harboring *INHBA*-enriched tumors being HPV-negative. Incidentally, RUNX2 was shown to upregulate the expression of *INHBA* in HNSCC (24), and recent reports confirmed an HPV-integration site upstream of *RUNX2* in close proximity to its promoter (25, 26). Consequently, a disruption in the expression of *RUNX2* by HPV-genome integration could influence the RUNX2/INHBA axis in HPV-positive OPSCC, leading to INHBA downregulation. Moreover, patients with HPV-negative OPSCC are known for high nicotine consumption throughout their lifetime. Ashour and colleagues showed that nicotine upregulates *INHBA* in chicken embryos (27). Hence, *INHBA* overexpression could be attributed to nicotine consumption.

INHBA affects cancer progression by regulating a plethora of oncogenic hallmarks. In breast cancer, INHBA has been shown to induce EMT and enhance the motility of malignant cells (28). The latter was also shown by Kang and colleagues to result from *INHBA* overexpression in prostate cancer cells (10). In addition, INHBA was found to promote cancer progression in lung adenocarcinoma by accentuating the proliferation of tumor cells (12). Li and colleagues showed that targeting INHBA in ovarian cancer cells is implicated in hindering the activation of stromal fibroblasts (29). Nonetheless, in OPSCC, the functional role of INHBA still needed to be elucidated. The enrichment of *INHBA* in tumors is shown in the current study to be implicated in the upregulation of EMT in HPV-negative OPSCC. We demonstrated that *INHBA* was expressed in UDSCC1, an HPV-negative OPSCC cell line. We could also show that the knockdown of *INHBA* made UDSCC1 cells less migratory and proliferative while simultaneously potentiated cell death. Its knockdown significantly decreased the EMT transcription factor *SNAI2* level, which stands as the downstream target by which INHBA initiates EMT in OPSCC. The latter was also shown to increase motility and promote mesenchymal phenotype in breast cancer cells (30). One potential mechanism by which *INHBA* knockdown hinders cell proliferation was described by Zhang and colleagues, showing downregulation of Cyclin D1 (*CCND1*) upon *INHBA* gene silencing (31). Another possibility is the upregulating of *SMAD2/3* by *INHBA*, as described in nasopharyngeal carcinoma (32) and pancreatic ductal adenocarcinoma (33). SMAD2/3, in turn, can interact with FOXO1/3/4, which are responsible for the upregulation of CDKN1A (34). Regarding apoptosis resistance, an interaction between SMAD2/3 and TP53 activates the transcription of *SERPINE1*, which in turn was shown to activate the anti-apoptotic PI3K/Akt pathway (35).

In OPSCC primary tumors, we could show a significant positive correlation between the expression of *INHBA* and nine previously described HNSCC stemness-related markers, with *CD44*, *ALDH1L2*, *HIF1A*, and *ZFP42* exhibiting the strongest association. We additionally confirmed the essentiality of *INHBA* for clonal expansion as its knockdown *in vitro* significantly hindered the capability of single UDSCC1 cells to survive, expand, and form colonies. Furthermore, we could show that the knockdown of *INHBA* attenuated the activity of the ALDH enzyme, a known marker of cancer stem cells (36). In line with our findings, Lonardo and colleagues showed INHBA to be vital for the ability of pancreatic cancer stem cells to self-renew and maintain their stemness (14). One possible mechanism is via *CD44*, which, in our study, was shown to have a strong positive correlation with *INHBA* expression. In head and neck cancer, CD44 was shown to inhibit the phosphorylation of GSK3β, which is required to maintain stem cell self-renewal (37).

The relationship between INHBA and tumor immune infiltrates was studied in various solid cancers to understand its impact on the tumor microenvironment. We could show that *INHBA* negatively correlates with CD8^+^ T cells in OPSCC primary tumors, which concurs with the findings by Pinjusic and colleagues demonstrating the indirect ability of INHBA to reduce CD8^+^ T cells tumor infiltration in melanoma *in vivo* models by downregulating the production of CXCL9/10 (38). In contrast, *INHBA* expression correlated positively with infiltration of CD8^+^ T cells in colorectal (39) and breast cancer (40). Furthermore, *INHBA* exhibited a negative correlation with the level of tumor-infiltrating B cells, which have a controversial role in cancer (41). Nonetheless, the mere ability of B cells to present antigens to CD4^+^ T cells in tumor-associated tertiary lymphoid organs (42) might be hindered due to a lower level of infiltration in *INHBA*-enriched OPSCC tumors. Previously, it was shown that *INHBA* and *CCL19* exhibit opposite modes of expression in colon cancer, with INHBA being upregulated while CCL19 is concurrently downregulated (43). As CCL19 is known to be a B-cell chemoattractant (44), this could possibly predate the low levels of B-cell tumor infiltration.

To our knowledge, the association between *INHBA* and M1 macrophage infiltration has not been reported previously. However, a positive correlation was disclosed between *INHBA* expression and macrophage infiltration level in breast (40) and cervical cancer (45). Incidentally, we showed a positive association between *INHBA* expression and M1 macrophage infiltration in OPSCC tumors. M1 macrophages are typically considered to be antitumor. However, Kainulainen and colleagues recently described a protumor M1 macrophage subtype in melanoma, accentuating invasive melanoma cells by upregulating the TNFα signaling via NFκB in tumor cells (23). Our study revealed that the resulting tumor-upregulated molecular signature is also associated with the level of M1 macrophages infiltrating OPSCC tumors.

Various miRNAs were identified as oncogenes and tumor suppressors, and their aberrant expression was implicated in the development and progression of various types of cancers (46). miRNAs were extensively researched in HNSCC and shown to hold a prognostic value (47). We previously showed that miRNA as exosomal cargo correlates with clinical parameters in HNSCC (48, 49) and can alter biological processes like EMT (50). In the current study, three putative miRNAs, miR-106a, miR-20b, and miR-9, were outlined as repressors of *INHBA* expression, prompting their HPV-dependent upregulation in OPSCC prognostically favorable. The role of miRNAs in cancer is controversial and highly dependent on cancer context and entity. Still, miR-106a was hitherto reported to be upregulated by HPV E7 and to enhance radiation sensitivity by downregulating the expression of the *RUNX3* axis in HNSCC (51). miR-20b, which belongs to the miR-106a-363 cluster, was associated with a better prognosis in HNSCC (52). miR-9 also, was shown to function as a tumor-suppressive miRNA in oral squamous cell carcinoma (OSCC), OPSCC, and nasopharyngeal carcinoma (NPC; refs. 53–55).

Recently, Xiao and colleagues revealed that 10 mmol/L of metformin, an oral antihyperglycemic used to treat type 2 diabetes mellitus, attenuates the expression of *INHBA* and inhibits the proliferation of colorectal cancer cells (56). An epidemiologic analysis based on three multicentric studies showed that metformin given for the treatment of diabetes is associated with longer overall survival of patients with HNSCC despite the negative prognostic impact of diabetes itself (57). In addition, it was demonstrated that the combination of metformin and cisplatin significantly improved the therapeutic effect of cisplatin in OSCC (58) and NPC (59). In contrast, another study found no significant prognostic impact of metformin in patients with HNSCC (60). The conflicted finding regarding the impact of metformin on HNSCC prognosis may be attributed to discrepancies in the HPV status of patients; *INHBA* enrichment in HPV-negative tumors, as shown in the current study, possibly renders the impact of metformin HPV-dependent. Interestingly, an analysis of samples from a metformin clinical trial in HNSCC showed that, following metformin treatment (dose of 1000 mg), cancer cell apoptosis was substantially higher in HPV-negative mucosal HNSCC tumor samples than in HPV-positive OPSCC tumor samples. Also, metformin-treated specimens displayed a significantly higher CD8^+^ effector T-cell infiltrate than specimens not treated (61). Hence, metformin in the neoadjuvant and adjuvant treatment of patients with HPV-negative OPSCC with *INHBA*-enriched tumors should be considered.

We revealed *INHBA* upregulation in HPV-negative OPSCC primary tumors compared with HPV-positive and established its oncogenic function by means of numerous functional assays. Yet, uncertainty exists regarding whether tumor development *in vivo* is slowed down by *INHBA* knockdown. It would be intriguing to look into patient-derived xenografts of INHBA-enriched tumors concurrent to the knockdown of *INHBA* and ultimately evaluate tumor progression.

In short, we found *INHBA* enriched in HPV-negative OPSCC tumors as a top-upregulated gene in the EMT pathway. We showed that the initiation of EMT by *INHBA* is mediated by the upregulation of *SNAI2* and *SNAI1*, rendering tumor cells more migratory. We could also show that the knockdown of INHBA attenuates proliferation and enhances cell death. We revealed that INHBA potentiates stemness and positively correlates with the expression level of stemness-related markers like *CD44*, *ALDH1L2*, *HIF1A*, and *ZFP42*. Furthermore, we outlined three miRNAs as putative repressors of INHBA, for which expression levels exhibited an inverse prognostic impact compared with *INHBA*. These findings delineate *INHBA* as an attractive therapeutic target to treat patients with HPV-negative OPSCC with *INHBA*-enriched tumors.

## Supplementary Material

Supplementary Table 1 Gene set enrichment analysis output

Supplementary Table 2 Differential expression analysis output

Supplementary Table 3 Correlation analysis between miRNAs and INHBA expression output

Supplementary Figure 1 E-Cadherin Expression upon INHBA knockdown. Representative western blot (of n = 3 replicates) showing E-Cadherin protein expression in Mock KD and INHBA KD1/KD2 at t24 and t48.
