# Supplementary figures and images for: INHBA is Enriched in HPV-negative Oropharyngeal Squamous Cell Carcinoma and Promotes Cancer Progression
Source: Cancer Res Commun. 2024 Feb 28;4(2):571–87. doi: 10.1158/2767-9764.CRC-23-0258 (PMC10901070; doi:10.1158/2767-9764.CRC-23-0258)

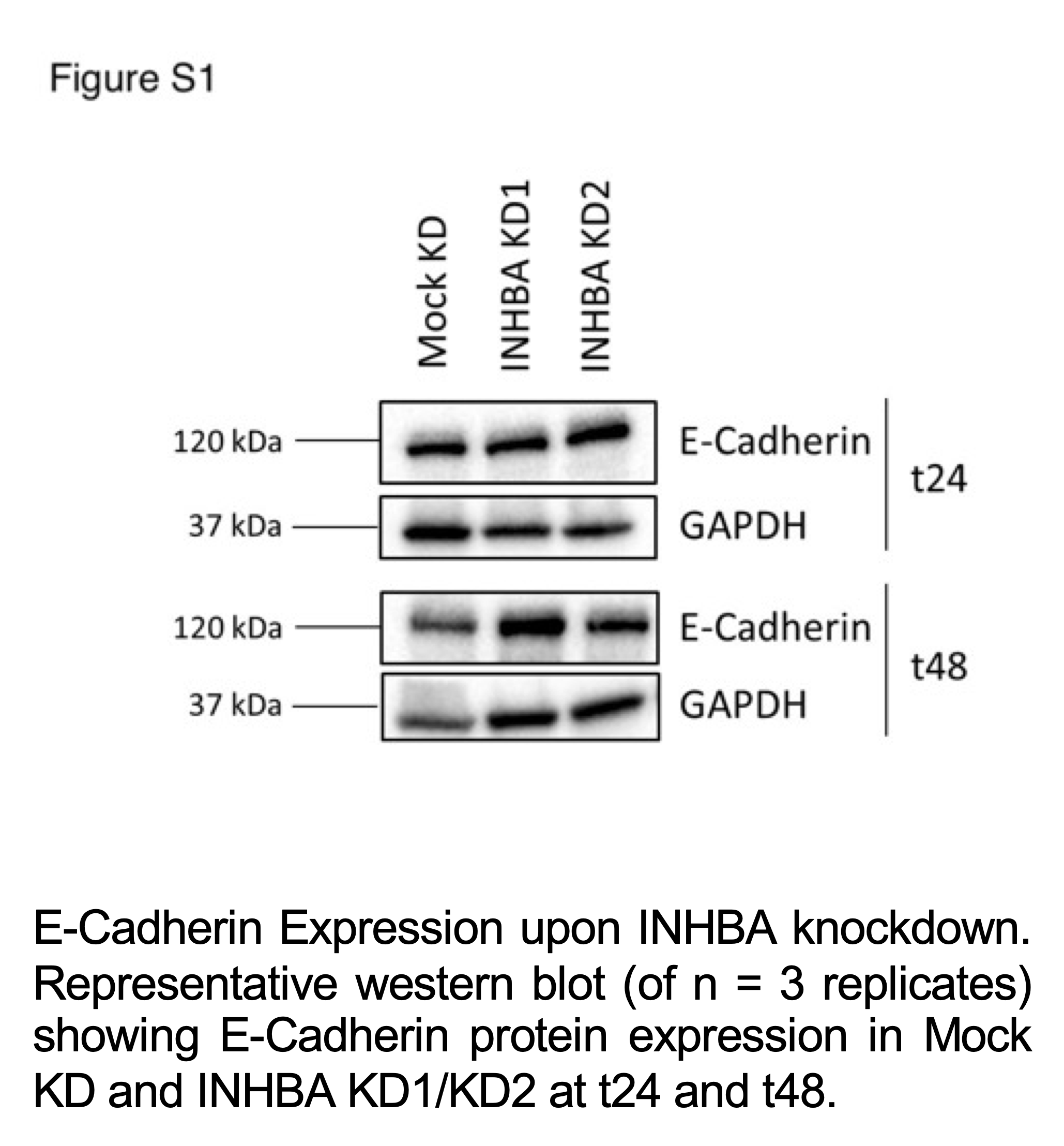

Supplement: Supplementary Figure 1 — E-Cadherin Expression upon INHBA knockdown. Representative western blot (of n = 3 replicates) showing E-Cadherin protein expression in Mock KD and INHBA KD1/KD2 at t24 and t48. [file crc-23-0258-s04.png]
